# Supplementary material for: IFN-Inducible SerpinA5 Triggers Antiviral Immunity by Regulating STAT1 Phosphorylation and Nuclear Translocation
Source: Int J Mol Sci. 2023 Mar 13;24(6):5458. doi: 10.3390/ijms24065458 (PMC10049297; doi:10.3390/ijms24065458)
Supplement: Supplementary file 1 [file ijms-24-05458-s001.zip › ijms-2267266-supplementary.pdf]

Supplemental Table 1. List of primers used in qRT-PCR analysis

Target	Forward primer (5' to 3')	Reverse primer (5' to 3')
h-SerpinA5	AATGCCCTTTTCACCGACCT	GCCAGGTACAGCGTCTTCAT
m-SerpinA5	TGGCTTCCCGCCGACACTCC	GAAGAAGACATTCTGACCAG
Ul-27	GCCTTCTTCGCCTTTCGC	CGCTGTGCCCTTCTTCTT
h-GAPDH	GTCAACGGATTTGGTCGTATTG	AAACCATGTAGTTGAGGTCAAT
h-IFN-α	TTTCTCCTGCCTGAAGGACAG	CTCATGATTTCTGCTCTGACA
h-IFN-β	AAAGAAGCAGCAATTTTCAG	CCTTGGCCTTCAGGTAATGCA
h-IFN-λ1	CTTCCAAGCCCACCCCAACT	GGCCTCCAGGACCTTCAGC
h-TNF-α	CTTCTCGAACCCCGAGTGAC	ATGAGGTACAGGCCCTCTGA
h-IL-1β	CAGAAGTACCTGAGCTCGCC	CATGGCCACAACAACTGACG
h-Mx1	GCCGGCTGTGGATATGCTA	TTTATCGAAACATCTGTGAAA

Supplemental Table 2. List of si-RNA sequences used in this study

Target	Forward primer (5' to 3')	Reverse primer (5' to 3')
h-SerpinA5-366	GCAGAAGGGACUUUACCUUTT	AAGGUAAAGUCCCUUCUGCTT
h-SerpinA5-820	GGUCGUGAUCAUGGUGAAUTT	AUUCACCAUGAUCACGACCTT
h-SerpinA5-1126	GCAGCUCGAGCUUUACCUUTT	AAGGUAAAGCUCGAGCUGCTT

Supplemental Figure 1 SrpinA5 is an IFN-Stimulated Gene

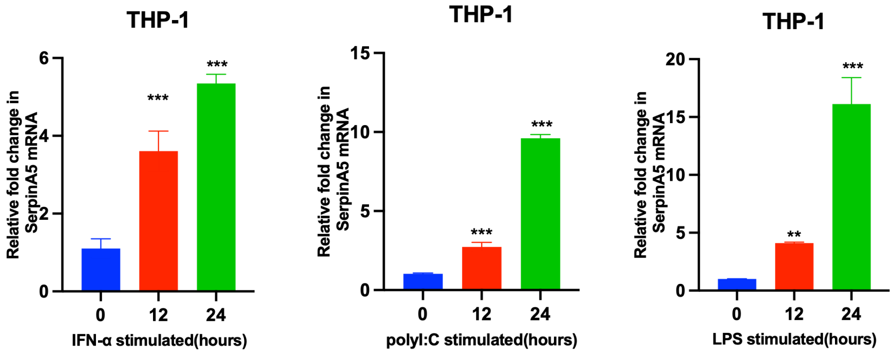

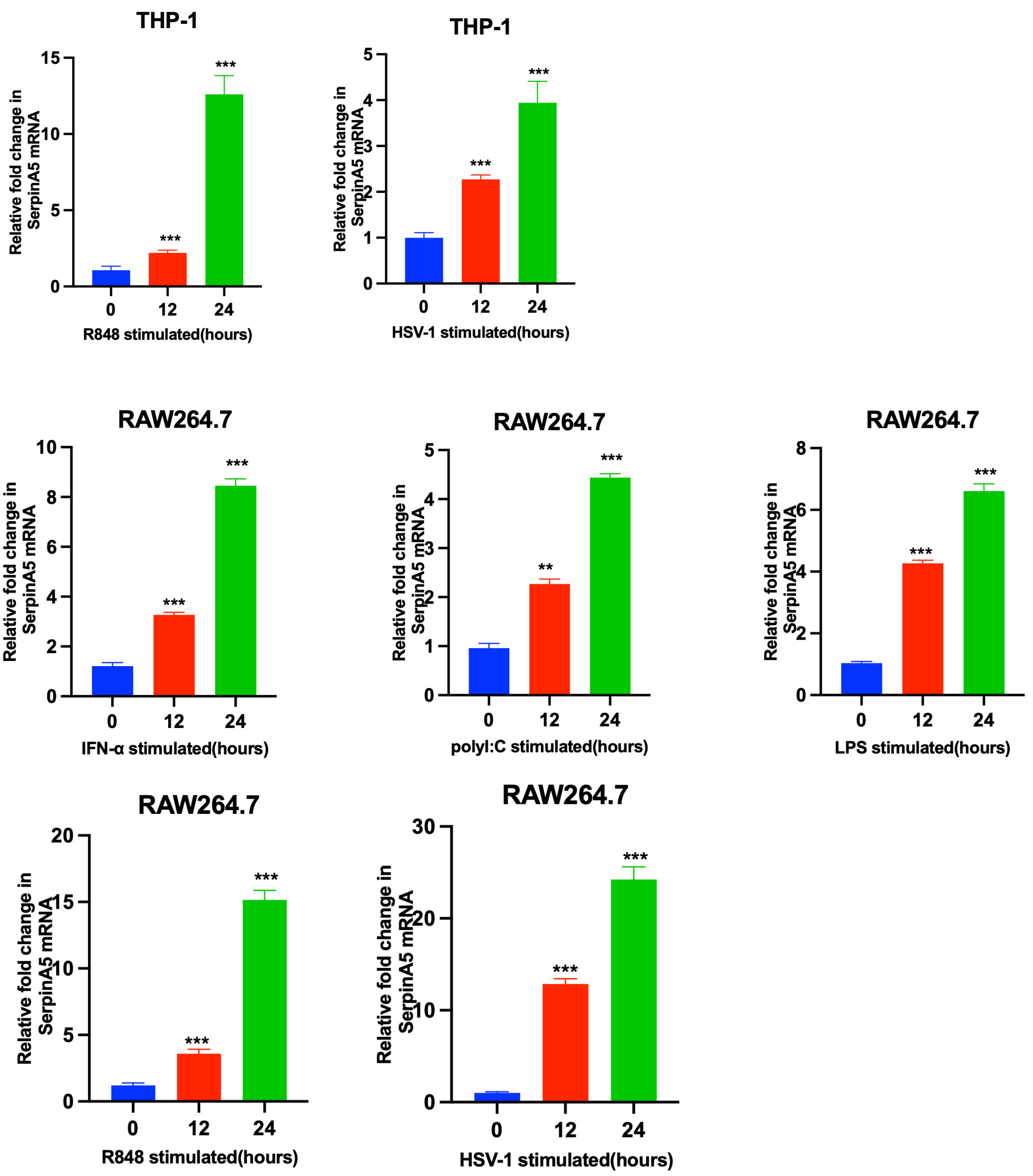

Figure1. THP-1 and RAW264.7 cells were stimulated with IFN-α (2000 U/mL), Poly I:C(25μg/mL), R848 (100 nM), and HSV-1 (MOI = 0.5) respectively, and then the expression of SerpinA5 were detected by qPCR respectively. The expression level of mRNA was normalized to the expression of GAPDH, and the data from at least triplicates were shown as the mean ± SD. * $p < 0.05$, ** $p < 0.01$, *** $p < 0.001$.

Supplemental Figure 2 Stable SerpinA5-overexpressed HEK-293T cell line

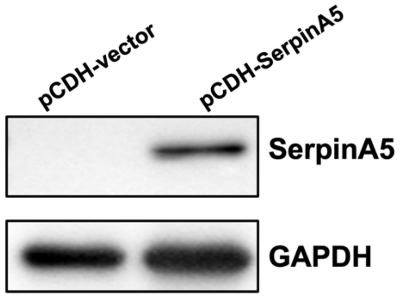

Figure2. SerpinA5 was inserted into pCDH-CMV-MCS-EF1-Puro vector to obtain SerpinA5 overexpressing plasmid pCDH-SerpinA5. pCDH-SerpinA5 lentivirus particles were obtained by co-transfecting pCDH-SerpinA5 with packaging plasmids (pMD2 and pAX2) into HEK293T cells. HEK-293T cells were infected with concentrated pCDH-SerpinA5 lentivirus particles and then were screened by puromycin to obtain stable SerpinA5-overexpressed HEK-293T cell line.
